# A computational model of circRNA-associated diseases based on a graph neural network: prediction and case studies for follow-up experimental validation

**DOI:** 10.1186/s12915-024-01826-z

**Published:** 2024-01-29

**Authors:** Mengting Niu, Chunyu Wang, Zhanguo Zhang, Quan Zou

**Affiliations:** 1 School of Electronic and Communication Engineering, Shenzhen Polytechnic University, Shenzhen, 518055 China; 2 School of Life Science and Technology, University of Electronic Science and Technology of China, Chengdu, China; 3 Faculty of Computing, Harbin Institute of Technology, Harbin, 150000 Heilongjiang China; 4 Hepatic Surgery Center, Tongji Hospital, Tongji Medical College, Huazhong University of Science and Technology, 1095 Jiefang Avenue, Wuhan, 430030 China; 5 Institute of Fundamental and Frontier Sciences, University of Electronic Science and Technology of China, No. 4 Block 2 North Jianshe Road, Chengdu, 610054 China; 6 Yangtze Delta Region Institute (Quzhou), University of Electronic Science and Technology of China, Quzhou, China

**Keywords:** circRNA, Disease, Biological networks, Graph Markov neural network, Variational inference, Biological experiment verification

## Abstract

**Background:**

Circular RNAs (circRNAs) have been confirmed to play a vital role in the occurrence and development of diseases. Exploring the relationship between circRNAs and diseases is of far-reaching significance for studying etiopathogenesis and treating diseases. To this end, based on the graph Markov neural network algorithm (GMNN) constructed in our previous work GMNN2CD, we further considered the multisource biological data that affects the association between circRNA and disease and developed an updated web server CircDA and based on the human hepatocellular carcinoma (HCC) tissue data to verify the prediction results of CircDA.

**Results:**

CircDA is built on a Tumarkov-based deep learning framework. The algorithm regards biomolecules as nodes and the interactions between molecules as edges, reasonably abstracts multiomics data, and models them as a heterogeneous biomolecular association network, which can reflect the complex relationship between different biomolecules. Case studies using literature data from HCC, cervical, and gastric cancers demonstrate that the CircDA predictor can identify missing associations between known circRNAs and diseases, and using the quantitative real-time PCR (RT-qPCR) experiment of HCC in human tissue samples, it was found that five circRNAs were significantly differentially expressed, which proved that CircDA can predict diseases related to new circRNAs.

**Conclusions:**

This efficient computational prediction and case analysis with sufficient feedback allows us to identify circRNA-associated diseases and disease-associated circRNAs. Our work provides a method to predict circRNA-associated diseases and can provide guidance for the association of diseases with certain circRNAs. For ease of use, an online prediction server (http://server.malab.cn/CircDA) is provided, and the code is open-sourced (https://github.com/nmt315320/CircDA.git) for the convenience of algorithm improvement.

**Supplementary Information:**

The online version contains supplementary material available at 10.1186/s12915-024-01826-z.

## Background

circRNA is a covalently closed RNA produced by back-splicing [1]. circRNAs are widespread in eukaryotes, evolutionarily conserved, tissue-specific, highly stable, and can accumulate in neural tissues [2, 3]. With rapid improvements in biological sequencing technology, multiple circRNA molecules, such as circNSUN2, CircHIPK3, and circNTNG1, have been discovered [4–6]. circRNA’s functions and its use as a biomarker and therapeutic target for various diseases, such as liver cancer and cervical cancer, are also increasingly being studied [7]. circRNAs play an important role in several diseases such as atherosclerotic vascular disease, nervous system disease, infectious disease, and cancer and are abnormally expressed in rectal cancer and pancreatic duct malignancies [2, 8]. For example, CDR1as is associated with miR-7, thereby affecting the occurrence and development of diabetes, metabolic diseases, brain developmental diseases, and cancer [9]. circRNA is involved in almost all human pathological, physiological, and other biological processes and may become a functional biomarker and therapeutic target for various diseases [10]. This would not only enable us to have a deeper understanding of circRNA but also provide us with a new research direction for the diagnosis and prevention of certain diseases.

To date, many predictors were proposed for predicting new associations from known circRNA-disease associations. Such methods include GMNN2CD [11], iCircDA-MF [12], PWCDA [13], iCDA-CGR [14], GCNCDA [15], KGANCDA [16], DWNN-RLS [17], SGANRDA [18], GATCDA [19], and RNMFLP [20]. Overall, these methods have greatly promoted research on association prediction.

The number of experimentally verified circRNA-associated diseases is still very small as it is time-consuming and laborious to reveal the role of circRNA molecules in various diseases through experiments such as biological tissues and cells [21]. Machine learning (ML) provides an efficient way to explore large-scale associations [22, 23]. circRNA candidate-associated disease prioritization by computational modeling will have powerful implications for guiding biological experiments and the comprehensive exploration of pathogenic mechanisms. To ensure the accuracy of predictions, researchers have been working on developing algorithms to identify potential circRNAs associated with diseases [24–26]. These predictors mostly make use of methods such as biological networks, recommendation algorithms, and ML. Among such methods, the computing model based on deep learning (DL) has recently become the most widely used algorithm [27, 28]. Cao et al. used a new computational method GGCDA involving an attention mechanism and graph convolutional network (GCN) for predicting associations [29]. He et al. found a disease-related circRNA-miRNA axis by using a GCN [30]. Wang et al. employed a two-layer convolutional neural network (CNN) for predicting association labels [31]. Wang et al. used the DL of the FastGCN to create the computational method GCNCDA and predict potential disease-related circRNAs [15]. He proposed a network embedding-based adaptive subspace learning method NSL2CD to predict potential and discover candidate genes associated with disease [18]. DL have achieved excellent results due to their powerful learning ability. In particular, GCN, which regards circRNA and disease as nodes in a graph and associations as the edges of the graph, perfectly combines the characteristics of networks and biology. However, when predicting, labels are predicted individually based on node feature representations, ignoring the dependencies between labels. In addition, feature inference and label propagation are independent of each other, making label propagation unable to fully utilize high-dimensional features. In previous studies, we proposed GMNN2CD, which combines variational algorithms and alternately performs feature inference and label propagation to construct a high-precision prediction algorithm. However, GMMN2CD ignores the biological data affecting circRNA-disease association and does not consider multisource biological data.

**Fig. 1:**
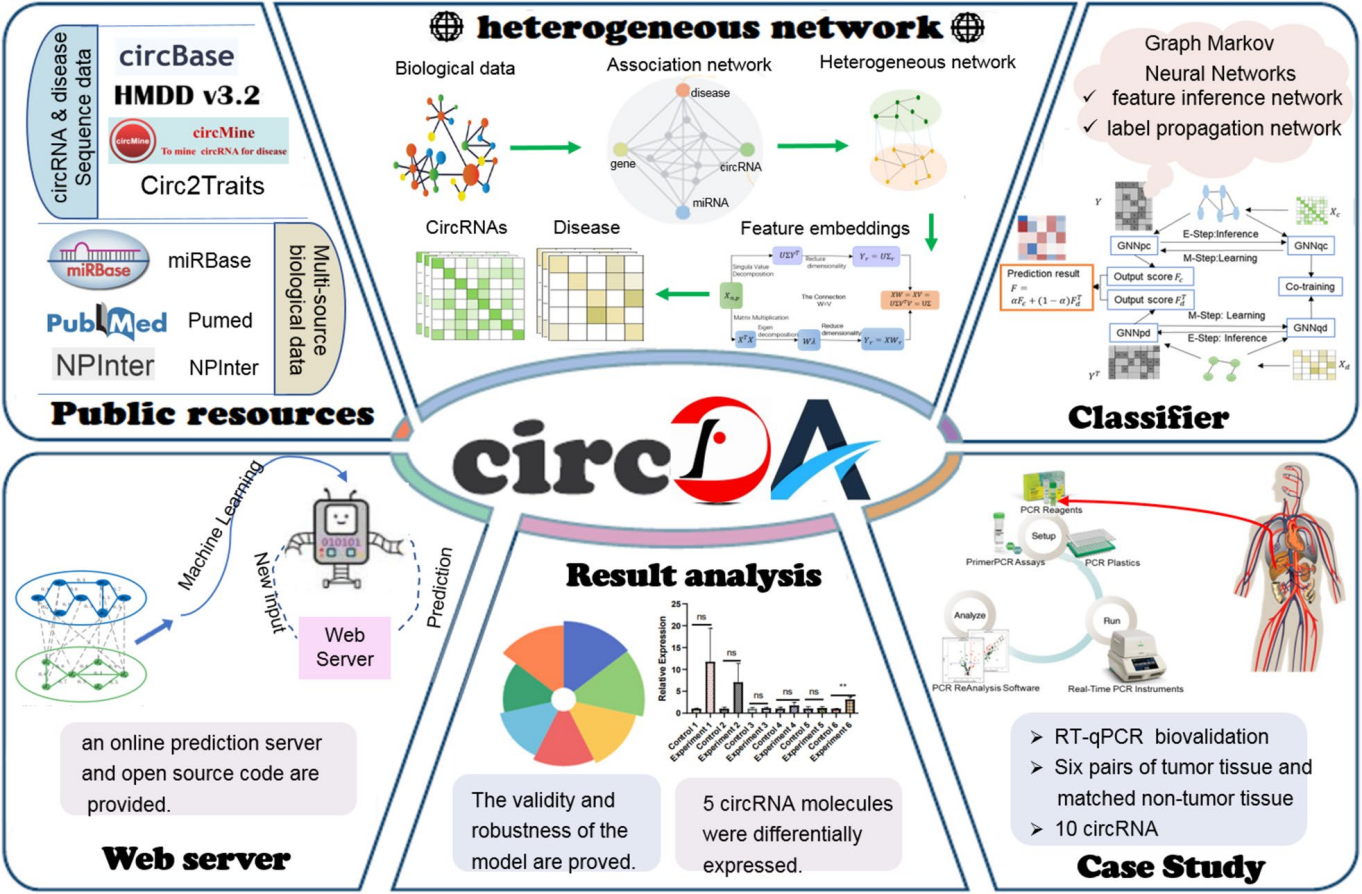
Structure and functionality of the online portal CircAD. CircAD builds a predictive model based on known experimentally verified associations between circRNAs and diseases and conducts experimental verification. CircDA includes dataset collation, heterogeneous network construction, classifier construction based on the Tumarkov neural network, HCC-based RT-qPCR experimental verification, and online server construction. CircAD provides users with an intuitive interface to browse, search, and predict circRNA-disease associations

This study proposes CircDA, a DL framework for predicting novel disease associations associated with circRNAs. The CircDA framework has several characteristics: (1) It introduces multisource data and combines rich omics data to construct a feature network related to circRNA and disease. (2) It uses matrix factorization (MD) to learn the embedding of the circRNA-disease association matrix and conFig.s, a convolutional network to learn deep feature representations. (3) The model combines a graph autoencoder and variational reasoning, using a feature reasoning graph network GNNq and a label propagation graph network GNNp; combined with a variational reasoning algorithm, the two networks GNNp and GNNq alternately learn features and propagate labels. (4) Based on ten circRNAs associated with HCC predicted by CircDA, a quantitative real-time PCR (RT-qPCR) case study was performed on human hepatocellular carcinoma (HCC) tissue samples. Through human biological experiments, it was found that five circRNAs out of ten circRNAs were significantly differentially expressed, which proved the predictive performance of CircDA and could further improve the functional research of circRNAs. (5) To facilitate the use of CircDA, we built an interactive and non-programming web interface. This can reduce the programming pressure on medical and biological workers. To facilitate in-depth research and improvements based on our work, the code is also open-sourced. This study shows that CircDA achieves higher accuracy than many state-of-the-art methods on evaluation data from benchmark data. Case studies demonstrate that CircDA can effectively predict unknown disease associations. The applicability and robustness of CircDA are demonstrated. The frame diagram of CircDA is shown in Fig. 1.

## Results

### CircDA performance

The main purpose of our study is to build a predictor with high accuracy in predicting diseases associated with circRNA molecules.

**Fig. 2:**
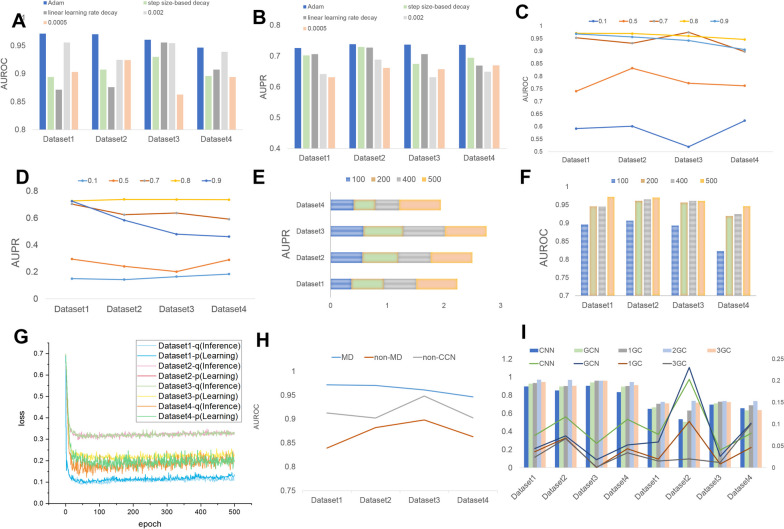
Performance analysis of our method, comparison of feature embedding strategies, and performance comparison of GMNN network models. **A**, **B** The AUROC and AUPR values of Adam’s LR scheme and the other four strategies under the four datasets, respectively. **C**, **D** The AUROC and AUPR values in the optimization process of a value under the grid optimization method, respectively. **E**, **F** The AUROC and AUPR values of CircDA during the epoch iteration, respectively. G The graph of the CircDA loss value in the epoch iterative training phase. **H** The comparison of the matrix factorization feature embedding strategy in CircDA with the other two strategies. **I** The comparison between GMNN network model and the other four models

We first optimize the learning rate (LR) according to the AUROC and AUPR. First of all, based on experience, we analyze the effect of three LR schemes (Adam, step size-based decay, linear learning rate decay) and fixed LRs (0.002, 0.0005). In Fig. 2A, B, the AUROC values obtained by the Adam method are 0.9716, 0.9426, 0.9465, and 0.9703, and AUPR values are significantly better than those of the other schemes. Therefore, Adam is chosen to be the LR of the model, and its initial value is empirically set to 0.001.

Furthermore, the hyperparameters *α* and *β* in the loss function Eqs. (11 and 12) lead to changes in performance. $\alpha \epsilon (\mathrm{0,1})$ represents the balance between circRNA and disease space. The grid search algorithm is used to find the optimal solution, and the search step size is 0.05. The AUROC and AUPR when the output $\alpha$ value is 0.1, 0.5, 0.7, 0.8, and 0.9 are displayed in Fig. 2C, D. As the $\alpha$ increases, the AUROC and AUPR of the model improve. The best performance is achieved with a value of 0.8. When the value of *α* beyond is 0.8, i.e., when $\alpha$ = 0.9, the performance of the CircDA begins to decline. After parameter optimization and comparison, the value of *α* is chosen to be 0.8.

Then, we analyzed the performance with different epochs (Fig. 2E, F) to verify the robustness of the CircDA and determine whether the CircDA exhibits overfitting. In Fig. 2E, F, as the epoch increased, the performance of the CircDA improved. However, AUROC and AUPR degrade when the epoch value is too large, which demonstrates the importance of the epoch. We then analyzed how the GNNp and GNNq loss values change as the epoch increased (Fig. 2G). As the epoch increased, the loss function curves of GNNp and GNNq of CircDA became stable after the initial non-fitting. This demonstrates the robustness of CircDA.

### Embedding enhances the predictive performance of CircDA

Feature embedding and deep representation are important components of our CircDA. So, we analyze the effectiveness of feature embedding and deep representation. To investigate the effectiveness of feature embedding, we compare the performance of the CircDA with that of models without MF (named non-MF) and models without CNN (non-CNN). Figure 2H shows that the performance of CircDA is optimal, higher than that of several other cases. First, the AUROC values of CircDA are 0.1185, 0.037, 0.064, and 0.0625 higher than those of non-MF, which proves that CircDA can learn the potential characteristics of circRNA and disease. Compared with non-CCN, the AUROC values of CircDA are 0.045, 0.0169, 0.0138, and 0.023, which proves that the deep features of circRNA and disease can be learned by using the convolutional network. By introducing feature embeddings, AUROC improves in four datasets, which shows that feature embeddings can improve predictive performance.

### Explore the optimal structure of our model CircDA

To quantify the importance of GMNNs for obtaining good predictions, we performed an ablation study by first changing the number of graph convolutional (GC) layers in the GMNN part of CircDA and then feeding the same features into standard CNN and GNN. These ablation studies were performed on four datasets to study generalizability. The AUROC histograms of the four datasets and the line graphs of the AUROC increase the value of CircDA compared with several other cases as shown in Fig. 2I. Reducing the number of GC layers per GMNNs will greatly reduce performance. However, the performance difference caused by this change is minimal. One possible explanation for this behavior is that through GC layers, CircDA can automatically learn and update weights to minimize the loss function value, and using more GC layers slightly improves the generalization ability of the highest classification level. However, this comes at the cost of a slight drop in accuracy in predicting lower classification levels. Then, compared with the common CNN and GNN models, the maximum and minimum values of AUROC and AUPR are 0.03, 0.0902, and 0.026, 0.02034. This demonstrates the performance of CircDA during learning.

### The proposed CircDA outperforms basic classifiers

**Fig. 3:**
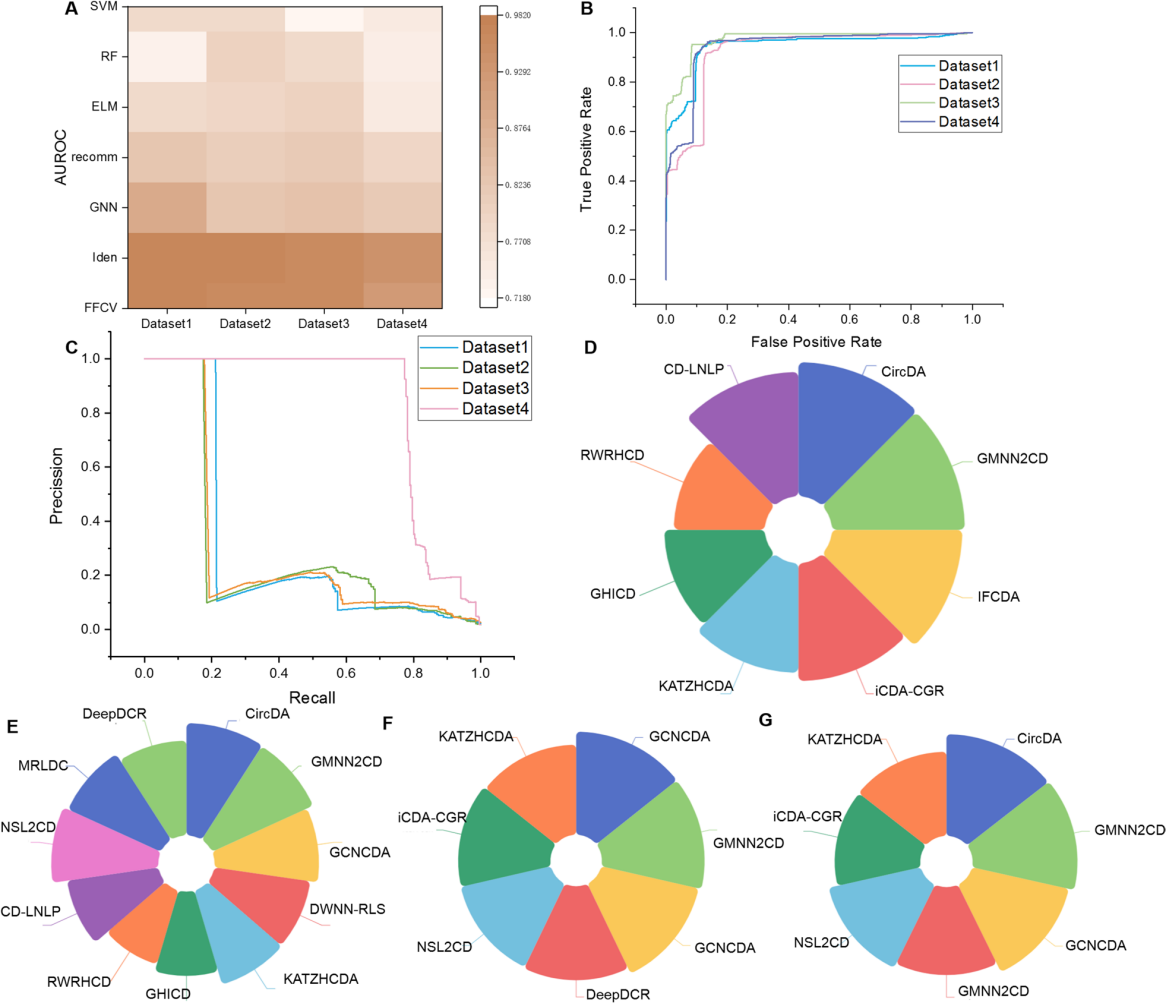
Performance comparison of CircDA with existing models. **A** AUROC values of our proposed CircDA and other basic classification methods on four benchmark datasets. **B**, **C** The ROC and PR curves of CircDA on the four datasets, respectively. **D**–**G** The AUROC values of CircDA and other existing methods on Dataset-1, Dataset-2, Dataset-3, and Dataset-4, respectively

In this section, we calculated the results of FFCV and independent test set validation (here, it is called Ide for short) of the CircDA and compared them with commonly used classifiers (extreme learning machine (ELM), random forest (RF), support vector machine (SVM) [32], and recommendation algorithm (here, it is called recomm for short)) [33]. Some important parameters of the algorithm use the default parameters built into the algorithm. The results are shown in Fig. 3A. First, the difference between the FFCV and Iden results of CircDA is very small, which proves the robustness of CircDA. Then, the AUROC of CircDA is markedly better than RF and SVM. Compared with RF and SVM, the AUROC of ELM, GNN, and recommendation algorithm has significantly improved, but it is also lower than CircDA. This also shows that CircDA can predict associations well.

### The proposed CircDA outperforms state-of-the-art models

In this section, based on Dataset-1 and Dataset-2, CircDA is compared with the most advanced models GMM2CD [11], DWNN-RLS [17], KATZHCDA [34], GHICD [12], RWRHCD [35], NCPCDA [36], CD-LNLP [37], CircDA-MF [12], CKA-GRTMF [36], and CKA-HGRTMF [36], and the quantitative AUROC results of each method are shown in Fig. 3D, E (the results of the compared methods are obtained from the literature). For Dataset-3 and Dataset-4, the algorithms GMNN2CD, KATZHCDA, iCDA-CGR, NSL2CD, DeepDCR, GCNCDA, and AUROC were reproduced using the code shared by the literature. GCNCDA based on deep learning fast learning and graph convolutional network; DWNN-RLS based on regularized least squares method; KATZHCDA based on enhanced induction matrix completion; CD-LNLP based on nearest neighbor label propagation; NSL2CD based on network embedding and subspace learning recognition; MRLDC integrated computing framework; DeepDCR based on deep learning. The results are shown in Fig. 3F, G.

In Fig. 3D–G, the CircDA method outperforms other advanced predicting methods on both datasets. CircDA performed best in FFCV, with AUROC values of 0.9716, 0.9703, 0.9607 and 0.9465. The performance of CircDA based on the GMNNs is significantly better than that of the traditional collaborative filtering recommendation algorithms ICFCDA, iCDA-CGR, DWNN-RLS, KATZHCDA, RWRHCD, CD-LNLP, and NSL2CD and better than that of MRLDC and DeepDCR, which are based on intelligent optimization algorithms and mathematical statistical model label learning methods. Since GMNNs can exploit object properties to propagate labels in a non-linear manner, they have a good ability to model label correlations. Compared with GCNCDA based on GCN, CircDA has obvious advantages. First, during the reasoning process, CircDA employs GNN to learn useful object-associated representations to improve reasoning ability. During the learning process, GNN is used to model local label dependencies. Furthermore, the predictive ability is further improved by including target attributes in the learned network, which demonstrates that CircDA can flexibly and efficiently add extra features to the learned network. Second, compared to GMNN2CD, our model CircDA has also been improved, which proves that it is necessary to consider biological network data. The effectiveness of the CircDA model built in this section is demonstrated by comparing it with existing methods.

### Case validation based on experimental results in the literature

To verify the performance of CircDA in predicting unknown associations, a case study of three cancers was conducted based on Dataset-4. Case studies in the literature include two types: case studies with known associations and case studies with unknown associations.

#### Case analysis of diseases with known associated circRNAs

The known associations are first applied to train CircDA. Next, disease-associated circRNAs are predicted using the trained model. After that, all candidate circRNAs are ranked according to the obtained prediction scores of all circRNAs. Finally, the predicted associations were validated by searching newly published literature.

**Fig. 4:**
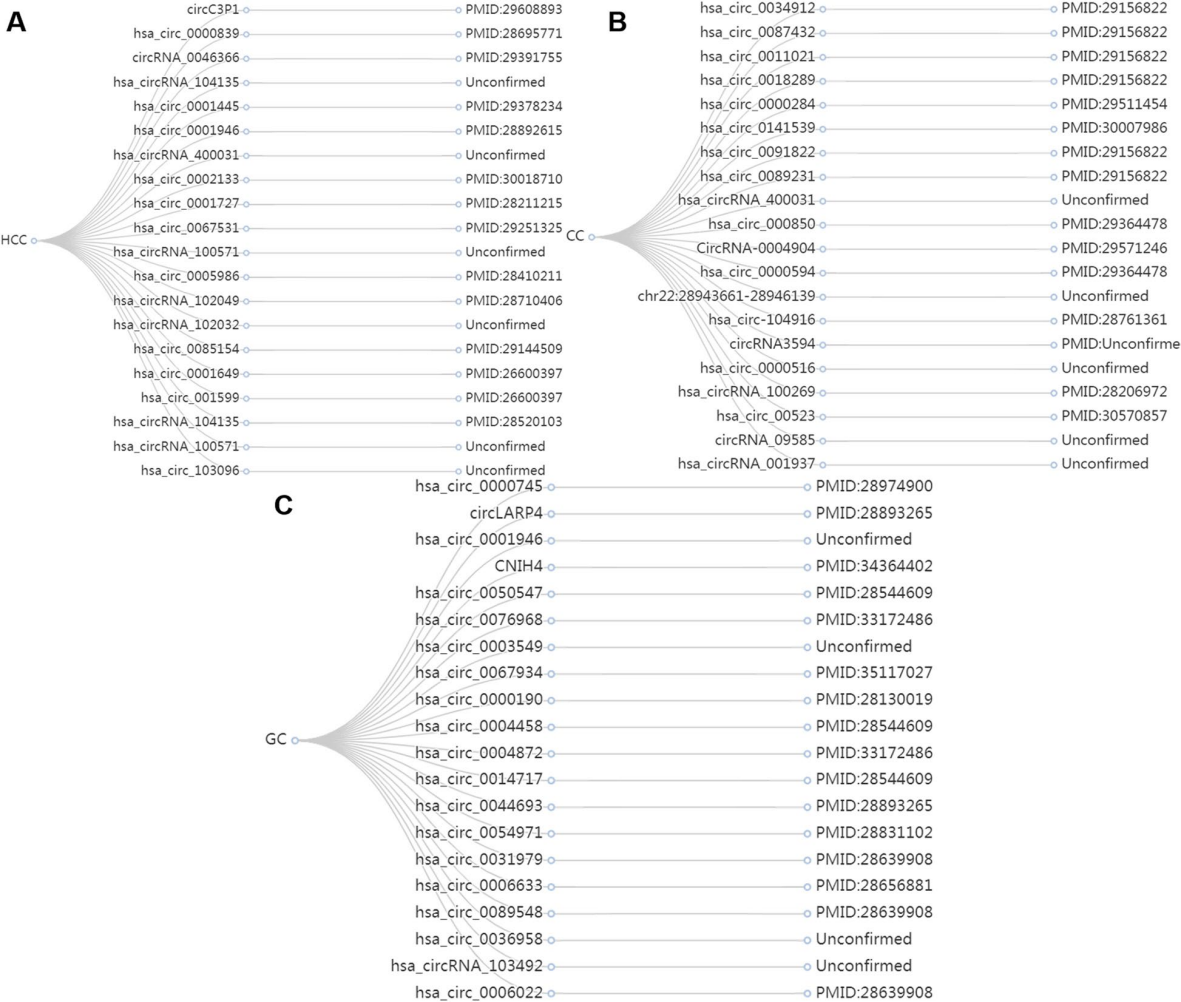
The prediction results of CircDA are verified based on the case analysis of the literature. **A**–**C** Twenty circRNAs predicted by CircDA on Dataset-2 related to HCC, CC, and GC, respectively

HCC is the most common malignancy worldwide. Accumulating evidence shows that circRNAs promote the growth of HCC cells. Therefore, we decided to verify the predictive performance of CircDA on HCC (Fig. 4A). Thirty circRNAs associated with HCC were included in the database, and 15 of the top 20 candidates were confirmed by the literature.

Cervical cancer (CC) is the most common gynecological malignancy. Studies have shown that circRNA plays a vital role in the occurrence of CC. Figure 4B lists the 20 circRNAs associated with CC with the highest prediction scores. CircDA predicted 16 out of 18 confirmed cervical cancers. Thus, we verified that CircDA has good predictive ability.

#### Case analysis of diseases with unknown associated circRNAs

To verify the predictive performance of CircDA for diseases without known associated circRNAs, taking gastric cancer (GC) as an example, we first deleted all circRNA data associated with gastric cancer in the database then used the remaining circRNA-disease association data to train CircDA, and finally used the trained model to predict gastric cancer. The association scores with circRNAs were sorted, and the predicted top 20 circRNAs were verified. The results are shown in Fig. 4C. Among the top 20 circRNAs, 16 have been verified, and the 4 unverified circRNAs are also expected to be verified in future biological experiments.

### RT-qPCR case validation of human HCC tissue samples

**Fig. 5:**
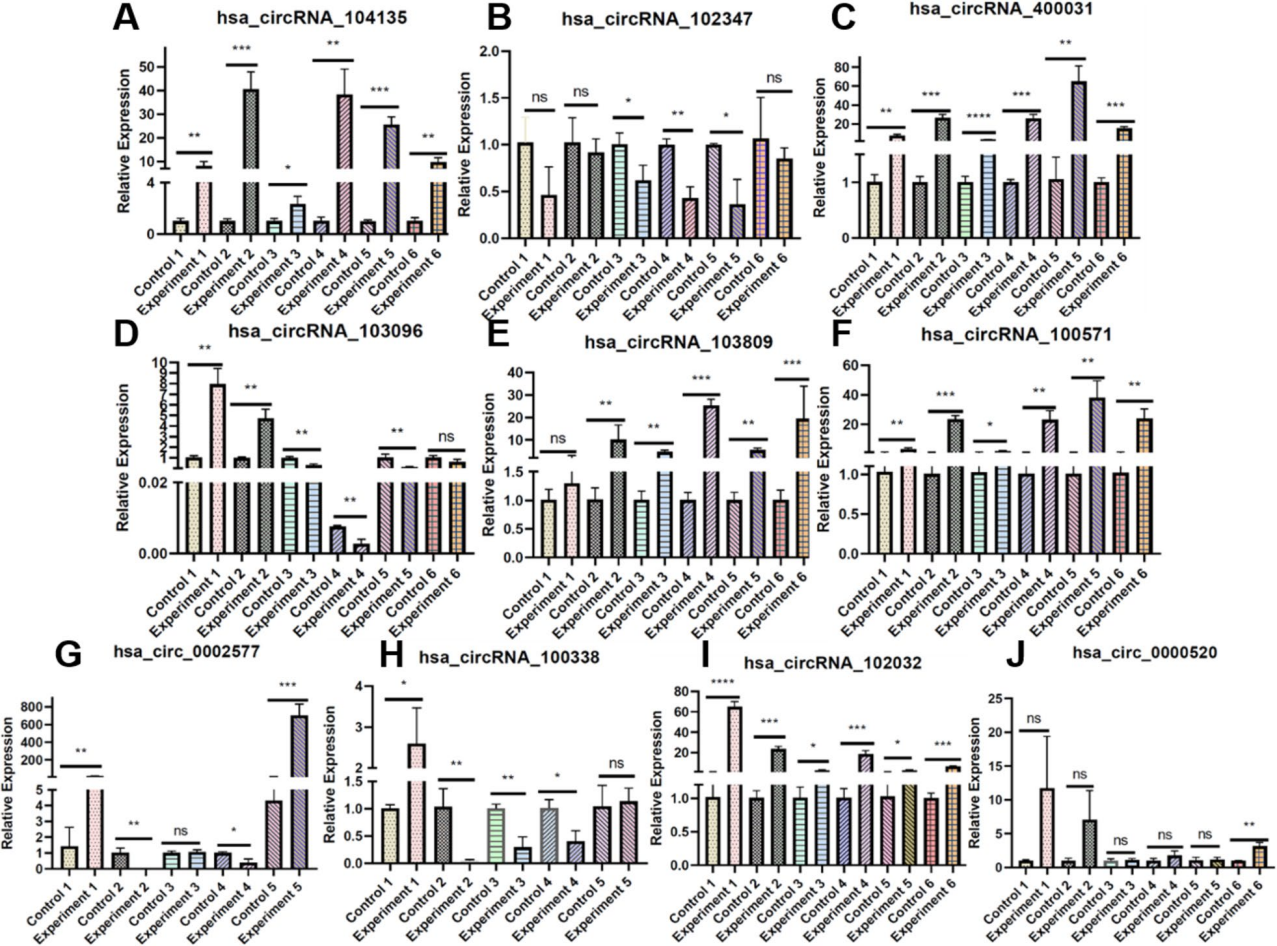
Experimental verification of ten circRNAs associated with HCC predicted by CircDA on human samples. **A**–**J** Ten circRNA molecules differential expression analysis in cancer and paracancerous tissues

For experimental verification, we removed the circRNAs included in the database and then selected the top ten circRNAs for RT-qPCR experimental expression analysis. Based on the prediction data of CircDA about HCC, and removing the confirmed circRNAs in Dataset-4, and the top ten circRNAs (hsa_circRNA_104135, hsa_circRNA_102347, hsa_circRNA_400031, hsa_circRNA_103096, hsa_circRNA_103809, hsa_circRNA_100571, hsa_circ_0002577, hsa_circRNA_100338, hsa_circRNA_102032, hsa_circ_0000520) were selected. These ten circRNA molecules are circRNAs not included in Dataset-4. Then, to identify specifically expressed circRNAs between HCC patients and normal individuals, we performed RT-qPCR on six tissue samples from HCC patients and control non-tumor tissue samples. We counted the expression of these ten groups of circRNAs in HCC and matched non-tumor tissue samples from patients. The results of paired sample mean/test analysis (Fig. 5) displayed that the expression of hsa_circRNA_104135, hsa_circRNA_400031, hsa_circRNA_103809, hsa_circRNA_100571, and hsa_circRNA_102032 in the six tissue samples were higher in HCC tissues than in paired paracancerous tissues. There was a statistically significant difference in the expression levels (*p* < 0.05). There was no significant difference between hsa_circRNA_102347 and hsa_circ_0000520 among the three tissue samples, and the other three showed higher expression levels in paracancerous tissues than in HCC tissue samples. hsa_circRNA_103096 had significant expression differences in the first five samples; the expression level of HCC tissue samples in the 1st and 2nd samples was higher than that of paracancerous tissues, and the opposite was true in the other samples. The RT-qPCR results of the sixth tissue samples of hsa_circ_0002577 and hsa_circRNA_100338 were abnormal, so this group of data is not counted. In the 1st and 5th samples of hsa_circ_0002577, the expression of the HCC tissue samples were higher than that of the paracancerous tissues, there was no significant difference in the 3rd sample, and the expression level of the paracancerous tissues was higher than that of the cancerous tissues in the other several samples. In the first sample of hsa_circRNA_100338, the expression level of the HCC tissue sample was higher than that of the paracancerous tissues, the situation was reversed in samples 2–4, and there was no difference in the 6th sample.

RT-qPCR analysis revealed that hsa_circRNA_104135, hsa_circRNA_400031, hsa_circRNA_103809, hsa_circRNA_100571, and hsa_circRNA_102032 maintained statistical significance between HCC cell lines and normal tissue samples. This proves that the prediction of the CircDA is reliable and can provide guidance for more circRNA disease function exploration, but more experimental verification may be needed. However, this model also provides a convenient way to subsequently verify the functions of specific biomolecules and provides guidance for the study of circRNA molecular functions.

### Web server

A web server with a friendly graphical user interface was created to share the constructed CircDA models among researchers. The interface takes circRNA sequences as input and returns the predicted diseases associated with it, where the recommended results are shown with the top five scores. At the same time, to facilitate local offline prediction, one can download the trained CircDA model and Python code. In addition, the website has a database function, and users can browse and download relevant data.

## Conclusions

In this work, based on rich multisource biological data, we develop a DL model combining variational algorithms and graph autoencoders. First, CircDA constructs heterogeneous features for multisource biological data. Then, CircDA uses the variable fusion feature inference network GNNq for feature inference and the label propagation network GNNp for label propagation. The two graph autoencoders are trained end-to-end using the variational EM algorithm. GMNN alternating training based on variational inference enhances CircDA’s ability to obtain efficient high-dimensional representation. CircDA proposed in this study achieved satisfactory results in circRNA-disease association prediction. Finally, through RT-qPCR experiments on HCC tissue samples and adjacent cancer tissues, five out of ten circRNA molecules were found to be differentially expressed, verifying the prediction ability of the model. At the same time, to better share CircDA, a web server was built. In addition to having a user-friendly interface and detailed online usage documentation, it hosts trained CircDA models and Python code that can be downloaded to your local computer for command-line use. CircDA can provide a reference for the prediction of unknown disease-related circRNAs and has certain usability. In addition, for the five circRNAs with obvious expression differences found in the RT-qPCR experiment, we will conduct literature research and then conduct a series of biological experiments on the most obviously different and unstudied circRNA to study their specific biological functions.

Moreover, there is still much room for improvement in circRNA-disease association research. For example, biological omics data are rich and diverse. We only considered miRNA and genes but did not consider multiomics data to construct a large-scale heterogeneous biomolecular association network with complete structure and biological significance. Therefore, exploring how multisource omics data affect the function of circRNA molecules may be an important direction for future work.

## Methods

### Human circRNA-disease associations

To evaluate the effectiveness of CircDA, benchmark datasets, commonly used as “gold standard” datasets, were used. To compare the effect of CircDA with those of state-of-the-art methods, we chose four benchmark datasets originally proposed by CircR2Disease (612 associations, 533 circRNAs, and 89 diseases) [38] and Circ2Disease (649 associations, 589 circRNAs, and 88 diseases) [39]. In addition, other circRNA-disease databases (circAtlas [40] and CircFunBase [41]) were included; in total, there were 930 associations (848 circRNAs and 110 diseases) and 2984 associations (2597 circRNAs and 67 diseases). CircR2Disease, Circ2Disease, circAtlas, and CircFunBase databases contain experimentally verified circRNA-related diseases, and we directly downloaded the corresponding data from the database. After unifying the circRNA names and deleting non-human circRNA/disease, 4 datasets were obtained, which were denoted as Dataset-1, Dataset-2, Dataset-3, and Dataset-4. That is, datasets of this study $S=\{{\text{Dataset}}-1, {\text{Dataset}}-2, {\text{Dataset}}-3, {\text{Dataset}}-4\}$. Datasets can be downloaded from web server (http://server.malab.cn/CircDA) and GitHub (https://github.com/nmt315320/CircDA.git).

We define an association matrix $A \epsilon {R}_{{c}_{m}*{D}_{n}}$ to represent the association of circRNAs with diseases, where $A({c}_{i},{d}_{j})=\{\mathrm{0,1}\}$.

1 $$A\left(c_i,d_j\right)=\left\{\begin{array}{ll}1,\;\mathrm{circRNA}\;\mathrm{is}\;\mathrm{associated}\;\mathrm{with}\;\mathrm{disease}\\0,\mathrm{circRNA}\;\mathrm{is}\;\mathrm{not}\;\mathrm{associated}\;\mathrm{with}\;\mathrm{the}\;\mathrm{disease}\end{array}\right.$$

CircRNA’s number is ${C}_{m}$, and the disease’s number is ${D}_{n}$.

### Human circRNA-miRNA-disease interaction network

In biological signaling regulatory network pathways, the interactions between circRNAs and miRNAs are often pathogenic. If a disease is associated with querying circRNA-bound miRNAs, then the disease may also be associated with circRNAs [42]. In this study, miRNA-circRNA interactions were collected from NPInter v4.0. Due to the limited experimentally verified circRNA-miRNA interactions, the classic algorithm miRanda was used to predict potential relationships with circRNA and miRNA. The data of circRNA and miRNA were extracted from circBase and miRBase databases, respectively. Associations between miRNAs and diseases, which were experimentally validated, were then collected from the HMDD3.2 database [43]. We collected 17,844 circRNA-miRNA associations (585 circRNAs and 640 miRNAs) and 1883 disease-miRNA associations (88 diseases and 462 genes).

### Human circRNA-gene-disease associations

Modern medicine has proved that human diseases are directly or indirectly related to genes. Gene mutations can cause a variety of diseases, and circRNAs regulate gene expression by competitively binding to miRNAs. Therefore, circRNAs that interact with disease-causing genes may also be associated with target diseases. Based on this, a heterogeneous circRNA-gene-disease network can be used to measure circRNA-disease associations. We downloaded circRNA-gene associations and disease-gene associations from http://cssb2.biology.gatech.edu/knowgene/search.html. We collected 487 circRNA-gene associations (585 circRNAs and 418 genes) and 74 disease-gene associations (88 diseases and 61 genes).

### Learning embedding features of circRNA and disease

Since there are few experimentally verified associations between circRNAs and diseases, the correlation matrix of these two variables is sparse. MD can well capture the shared and complementary information of different data sources, has the ability to resist noise and data heterogeneity, and can reduce the complexity of high-dimensional data. A high-dimensional matrix can be decomposed into two low-rank matrices whose product is close to the original correlation matrix [17].

Based on the association matrix A, this paper uses MD by expressing circRNA and disease embedding matrices *C* and *D* with latent factors. Then, the value of *CD*^*T*^ approximates the association matrix *A*, which is expressed as:

2 $$A\approx {CD}^{T}$$

where $C\in {R}_{m\times k}$, the *i*th row is the embedding of circRNA *Ci*; *D*∈*Rn*×*k*, and the *j*th row is the embedding of disease *Di*.

Using statistical learning theory, by constructing an objective function so that the total approximation error should be as small as possible, the two embedding matrices *C* and *D* obtained satisfy formula (2). Then, to avoid overfitting, a regularization term *L* is added to the objective function. Therefore, the objective function is defined as:

3 $$\underset{C,D}{{\text{min}}}(\frac{1}{2}{\Vert A-{CD}^{T}\Vert }_{F}^{2}+\alpha {\Vert L\Vert }_{F}^{2}+{\alpha \Vert D\Vert }_{F}^{2})$$

The update process for *C* and *D* is as follows:

4 $$\underset{D\in {R}^{n\times k}}{{\text{min}}}{J}_{1}\left(D\right)=(\frac{1}{2}{\Vert A-{CD}^{T}\Vert }_{F}^{2}+\alpha {\Vert D\Vert }_{F}^{2}$$

5 $$\underset{C\in {R}^{m\times k}}{{\text{min}}}{J}_{2}\left(C\right)=(\frac{1}{2}{\Vert A-{CD}^{T}\Vert }_{F}^{2}+\alpha {\Vert C\Vert }_{F}^{2}$$

Then, based on a layer of CNN, the latent features obtained by the MF are mapped to different spaces to obtain feature combinations.

### CircDA with graph Markov neural networks (GMNN) for circRNA-disease association predictions

CircDA uses GMNN to build prediction algorithms. GMNN is a DL algorithm that combines feature inference and label propagation. Variational inference and DL methods were used to propose a structure based on CircDA [44]. Variational inference includes E-step and M-step. The GMNN framework is proposed by our work in 2022 [11].

To predict circRNA-disease associations, we define a graph $G=(N, E,{f}_{N})$, where *N* is the node set, *E* is the edge set, and ${f}_{N}$ is the node attribute set. The goal is to get unknown labels ${y}_{U}$ based on some of the known labels ${y}_{C}$($C\in N$). The CircDA framework includes two graph autoencoders, namely, GNNq for feature reasoning and GNNp for label propagation and uses the E-step and M-step of the variational inference algorithm to alternately execute GNNq and GNNp to achieve effective optimization.

The variational inference algorithm is implemented by minimizing the losses *Lq* and *Lp* of GNNq and GNNp, respectively. As in other variational GCNs, *Lq* consists of the reconstruction error *Lqr* and divergence ${L}_{KL}$, and *Lp* consists of the reconstruction error *Lpr* and popularity loss ${L}_{m}$. *Lq* and *Lp* are defined as follows:

6 $${L}_{q}={L}_{qr}+{L}_{KL}$$

7 $${L}_{qr}=\frac{1}{2}{|\left|x-{x}{\prime}\right||}_{F}^{2}$$

8 $${L}_{KL}=-\sum\nolimits_{i,j}\frac{1}{2}(1+2{\text{log}}{\sigma }_{i,j}-{\mu }_{ij}^{2}-{\sigma }_{ij}^{2})$$

9 $${L}_{p}={L}_{pr}+{L}_{m}$$

10 $${L}_{pr}=-\sum\nolimits_{i,j}{Y}_{ij}{\text{log}}{F}_{ij}$$

After calculating the losses *Lp* and *Lq* of the two graph autoencoders, it is of importance that integrate information of the circRNA space and the disease space. Therefore, we cotrain GNNqc and GNNqd. Defining *Zc* and *Zd* as the representations learned in circRNA and disease space, respectively, the cotraining loss *Lc*, which measures the performance of cotraining, is defined as follows:

11 $${L}_{q}=\mathrm{\alpha }{L}_{qc}+\left(1-\alpha \right){L}_{qd}+\beta {L}_{c}$$

where ${L}_{qc}$ and ${L}_{qd}$ denote the losses of GNNqc and GNNqd calculated by Eq. (6), respectively. Since *Lm* and *Lc* depend on the computation of GNNqc and GNNqd, the effects of manifold constraints and co-training are related to the effectiveness of GNNq in capturing representations. Therefore, the hyperparameter *β* should be increased as the training progresses to enhance the robustness of representation learning and the convergence of the EM algorithm. Therefore, in CircDA, we set *β* = e/epoch in the *e*th epoch, where epoch stands for the total number of epochs. Likewise, the total loss of GNNp is:

12 $${L}_{p}=\mathrm{\alpha }{L}_{pc}+\left(1-\alpha \right){L}_{pd}$$

where ${L}_{pc}$ and ${L}_{pd}$ denote the losses of GNNpc and GNNpd, respectively.

After the training, *F*_*c*_ (${F}_{c}\epsilon {\mathbb{R}}^{m\times n}$) and *F*_*d*_ (${F}_{d}\epsilon {\mathbb{R}}^{m\times n}$) are the outputs of GNNpc and GNNpd, respectively. ${F}_{c}$ and ${F}_{d}$ are low-rank matrices computed by autoencoders:

13 $${\text{rank}}\left(a{F}_{c}+b{F}_{d}\right)\le {\text{rank}}\left({F}_{c}\right)+{\text{rank}}\left({F}_{d}^{T}\right),\forall a,b$$

Then the final output result *F* is:

14 $$F=\alpha {F}_{c}+(1-\alpha ){F}_{d}^{T}$$

### Performance evaluation

We use fivefold cross-validation (FFCV) to evaluate the performance of CircAD. Among them, the evaluation indicators are the area under the receiver operating characteristic curve (AUROC) and the area under the precision-recall curve (AUPR) [45–50]. AUROC is widely employed to evaluate the ability of binary classifiers, and the horizontal axis is the vertical axis. AUPR is the area under the PR curve, which is plotted with a true positive rate (TPR) as the horizontal axis (a higher TPR indicates that the model can predict more data) and precision as the vertical axis (a higher precision indicates that the correctness of the predicted samples is higher).

### HCC specimens

After the prediction model CircDA is constructed, we predict the circRNAs related to HCC. According to the ranking of the prediction results, remove the circRNAs included in Dataset-4 and get the top ten circRNA molecules. These circRNAs may have been underwritten by experiments and databases, or they may not be verified by experiments. We then collected human HCC tissue samples for RT-qPCR validation.

In 2022, researchers from the first hospital of Tongji Medical College of Huazhong University of Science and Technology in Wuhan collected cancer tissues and paired adjacent non-cancerous tissues from six patients with primary HCC. Six pairs of HCC samples (tumor tissue and matched non-tumor tissue) were used for circRNA microarray analysis. All tissue samples were taken during the operation and immediately frozen at 80 °C for subsequent experiments. Pathologists evaluated patients’ liver specimens and determined their clinical stage of HCC according to the BCLC classification. The following HCC patients were excluded: (1) patients aged 18 or 70 or without full capacity for civil conduct; (2) patients with a history of anticancer radiotherapy or chemotherapy, biology, immunization, or traditional Chinese medicine before surgery; (3) postoperative patients with incomplete follow-up data; and (4) patients with a history of other organ malignancies or systemic immune diseases. Written informed consent was obtained from each participant prior to tissue collection. The study protocol was approved by the Clinical Research Ethics Committee of Tongji College, Huazhong University of Science and Technology, Wuhan.

### RNA extraction, cDNA synthesis, and RT-qPCR

Following the manufacturer’s instructions, we extracted total RNA from cells using TRIzol Reagent (Invitrogen, Carlsbad, CA, USA) and treated with RQ1 DNase (Promega, Madison, WI, USA) to remove DNA. The specific steps of RNA extraction are as follows: (1) take the cell pellet, add 1 ml TRIzol to fully homogenize, and let stand at room temperature for 5 min; (2) add 0.2 ml of chloroform, shake vigorously for 15 s, and let stand for 3 min; (3) centrifuge at 4 °C, 12,000 rpm × 10 min, and take the supernatant; (4) add 0.5 ml of isopropanol, mix well, and let stand on ice for 20–30 min; (5) centrifuge at 4 °C, 12,000 rpm × 10 min, and discard the supernatant; (6) add 1 ml of 75% ethanol, wash the precipitate at 4 °C, 7500 rpm × 5 min, and discard the supernatant; and (7) dry at room temperature for about 5 min and add an appropriate amount of RNase-free H_2_O to dissolve.

Following the kit manufacturer’s instructions (TOYOBO Life Science, Shanghai, China), we used the ReverTra Ace qPCR RT Kit to perform reverse transcription reactions and measure the gene expression levels. Among them, each tissue sample was subjected to RT-qPCR amplification in triplicate. First, 1 μg of total RNA was reverse-transcribed into cDNA, and the steps and system were carried out according to the instructions. Then, using cDNA as a template, RT-qPCR internal reference gene actin primers were used for RT-qPCR amplification (the sequences and primer sequences of ten circRNAs included within its Additional files 1 and 2) to verify the quality of cDNA. The reaction conditions of RT-qPCR include three kinds: cycle at 95 °C for 1 min, cycle at 95 °C for 15 s, and cycle at 60 °C for 30 s. The final results were calculated relative gene expression as 2^−ΔΔCt^ and normalized.

### Supplementary Information

**Additional file 1:** **Table S1.** The sequences of 10 circRNAs.

**Additional file 2:** **Table S2.** The primer sequences of 10 circRNAs.

## Data Availability

All code and data generated or analyzed during this study are included in this published article, its additional files, and publicly available repositories: Zenodo (https://zenodo.org/record/8079147 [51]) and GitHub (https://github.com/nmt315320/CircDA.git [52]).

## Abbreviations

circRNAs: Circular RNAs
ML: Machine learning
DL: Deep learning
GMNN: Graph Markov neural network algorithm
HCC: Human hepatocellular carcinoma
RT-qPCR: Quantitative real-time PCR
GCN: Graph convolutional network
CNN: Convolutional neural network
MD: Matrix factorization
FFCV: Fivefold cross-validation
AUROC: Area under the receiver operating characteristic curve
AUPR: Area under the precision-recall curve
TPR: True-positive rate
LR: Learning rate
RF: Random forest
ELM: Extreme learning machine
SVM: Support vector machine
GC: Graph convolutional
CC: Cervical cancer
GC: Gastric cancer
