# Supplementary material for: A computational model of circRNA-associated diseases based on a graph neural network: prediction and case studies for follow-up experimental validation
Source: BMC Biol. 2024 Jan 29;22:24. doi: 10.1186/s12915-024-01826-z (PMC10823650; doi:10.1186/s12915-024-01826-z)
Supplement: Supplementary file 1 — Additional file 1: Table S1. The sequences of 10 circRNAs. [file 12915_2024_1826_MOESM1_ESM.docx]

Table S1. The sequences of 10 circRNAs

| CircRNA | Sequence |
| --- | --- |
| hsa_circRNA_104135 | GTCAGATGTCATGTAATCCTTCCTTTGGTGGCATCGGAAAGGGACATTTAATGAGGGAAGTAGATGCCTTGGATGGCCTGTGTTCTCGCATCTGTGACCAGTCTGGTGTACATTATAAAGTATTAAACCGGCGTAAGGGACCAGCTGTGTGGGGTCTGAGAGCTCAGATTGATAGGAAACTCTATAAACAGAACATGCAGAAAGAAATCTTGAATACACCACTGCTTACTGTTCAGGAGGGAGCTGTAGAAGATCTTATTCTTACAGAACCAGAGCCTGAACACACTGGGAAATGCCGTGTCAGTGGGGTTGTTTTGG |
| hsa_circRNA_102347 | CCAAACCAAACAGGAAGCTTACTTTTCTCTACCTAGCCAATGATGTCATACAGAACAGCAAGAGGAAGGGGCCAGAGTTTACAAAAGATTTTGCACCAGTTATAGTGGAGGCTTTTAAGCATGTTTCAAGTGAAACTGATGAAAGTTGTAAGAAGCACCTTGGAAGAGTGTTATCTATTTGGGAAGAAAGGTCTGTTTATGAAAATGATGTATTAGAACAACTTAAACAAGCTCTGTATGGTGATAAGAAGCCTAGGAAGCGAACTTATGAACAGATAAAGGTGGATGAAAATGAAAACTGTTCCTCTCTGGGATCTCCAAGTGAACCACCACAGACTCTAGATCTCGTTAGAGCATTACAAGATCTGGAAAATGCAGCCTCAGGTGATGCAGCAGTTCATCAGAGGATAGCTTCTTTACCTGTTGAAGTCCAAGAAGTATCTCTATTAGATAAAATAACAGATAAAGAATCTGGAGAAAGGCTTTCCAAAATGGTAGAGGATGCGTGTATGTTGCTGGCAGATTACAATGGCAGATTGGCGGCAGAAATAGATGATAGAAAGCAACTCACTCGAATGTTAGCAGATTTTCTTCGTTGTCAAAAGGAAGCCCTTGCAGAGAAAGAGCATAAATTGGAA |
| hsa_circRNA_400031 | CTGCTGTGAGGCCAGGGTCCAGGGGCAGCCTGGAGGGGAGCACAGTGGTCTTGAGACGCAGCCTCACAAAGCATAGCCACAGGACCTCTCCCTTGGGCCCTAGCACCTGCCTGGGCACAGAGGCAAGGAAGAGCCTCTGAGACCCCTCCTTCCTGTCCCACAGGACAGGAAATGCTCAGAGTTGCCAGGGGACCTGGGCAAAGACTCAAAGCTAACAAGTGACAGAAATGGGACTTGAGCCAGACCTTTTGACTCCAAGTCCAGCACTCTATCCCCCTCTCCCATGCACCTCCTCTCCTCCTGTCTTTCTCCTCCTTTCTGCGTATTATGAGGTGCCAAGACCTGATATAGGGGATGGAGGTAAAAAGAGATGGGGTGAGAAGCTGCAGCCCCTCCTCCC |
| hsa_circRNA_103096 | TGGAAGGAGGCAAAACCGGAAGACCTTATGGATTCAAAACTTAGATGTGTGTTTGAATTGCCAGCAGAGAATGATAAACCACATGATGTAGAAATAAATAAAATTATATCCACAACTGCATCAAAGACAGAAACACCAATAGTGTCTAAGTCTCTGAGTTCTTCTTTGGATGACACCGAAGTTAAGAAGGTTATGGAAGAATGTAAGAGGCTGCAAGGTGAAGTTCAGAGGCTACGGGAGGAGAACAAGCAGTTCAAG |
| hsa_circRNA_103809 | CCCTTACGTCGTCCTGACTCATCTGATGACCGTTATGTAATGACAAAACATGCCACCATTTATCCAACTGAAGAGGAGTTACAGGCAGTTCAGAAAATTGTTTCTATTACTGAACGTGCTTTAAAACTCGTTTCAGACAGTTTGTCTGAACATGAGAAGAACAAGAACAAAGAGGGAGATGATAAGAAAGAGGGAGGTAAAGACAGAGCTTTGAAAGGAGTTTTGCGAGTGGGAGTATTGGCAAAAGGATTACTTCTCCGAGGAGATAGAAATGTCAACCTTGTTTTGCTGTGCTCAGAGAAACCTTCAAAGACATTATTAAGCCGTATTGCAGAAAACCTACCCAAACAGCTTGCTGTTATAAGCCCTGAGAAGTATGACATAAAATGTGCTGTATCTGAAGCGGCAATAATTTTGAATTCATGTGTGGAACCCAAAATGCAAGTCACTATCACACTGACATCTCCAATTATTCGAGAAGAGAACATGAGGGAAGGAGATGTAACCTCGGGTATGGTGAAAGACCCACCGGACGTCTTGGACAGGCAAAAATGCCTTGACGCTCTGGCTGCTCTACGCCACGCTAAGTGGTTCCAGGCTAGAGCTAATGGTCTGCAGTCCTGTGTGATTATCATACGCATTCTTCGAGACCTCTGTCAGCGAGTTCCAACTTGGTCTGATTTTCCAAGCTGG |
| hsa_circRNA_100571 | GTCTCTGTTCTAGGATGTCCCGACCCAGTGGTGCATGAGATCGCCTATCAGTACGGAAAAAATGTAGGAATAGCTTTTCAGGTTAGTATGCTTTTTATTTGTAAGAATGGTGGCGTAGTGATACAGTCAGCATTCTCCCCTAGTGTGTAATCGTCAAAATAGTAAGAACGATGGCAGCAGTGTTGGCATGGCGGTGCTCCTTACATCCCATTTTTCCTTTTGCCAGCTAATAGATGATGTATTGGACTTCACCTCGTGTTCTGACCAGATGGGCAAACCAACATCAGCTGATCTGAAGCTCGGGTTAGCCACTGGTCCTGTCCTGTTTGCCTGTCAGCAG |
| hsa_circ_0000520\|NR_002312\|RPPH1 | GGAAGCTCATCAGTGGGGCCACGAGCTGAGTGCGTCCTGTCACTCCACTCCCATGTCCCTTGGGAAGGTCTGAGACTAGGGCCAGAGGCGGCCCTAACAGGGCTCTCCCTGAGCTTCGGGGAG |
| hsa_circ_0002577\|NM_025160\|WDR26 | GTATCTGATGTGTAGCCATGCAGAAGACCTACGTGCAAAAGCAGAATGGGAAGGCAAAGGGACAGCTTCCCGATCTAAACTATTGGATAAACTTCAGACCTATTTACCACCATCAGTGATGCTTCCCCCACGGCGTTTACAGACTCTCCTGCGGCAGGCGGTGGAACTACAAAGGGATCGGTGCCTATATCACAATACCAAACTTGATAATAATCTAGATTCTGTGTCTCTGCTTATAGACCATGTTTGTAGTAGGAGGCAGTTCCCATGTTATACGCAGCAGATACTTACGGAGCATTGTAATGAAGTGTGGTTCTGTAAATTCTCTAATGATGGCACTAAACTAGCAACAGGATCAAAAGATACAACAGTTATCATATGGCAAGTTGATCCG |
| hsa_circRNA_100338/hsa_circ_0000130 | GAACCACGTGAATGTTGAGGGGGCGACACACAAGCAGGTGGTGGACCTGATTCGAGCAGGCGAGAAGGAATTGATCTTGACAGTGTTATCTGTACCTCCTCATGAGGCAGATAACCTAGATCCCAGTGACGACTCGTTGGGACAATCATTTTATGATTACACAGAAAAGCAAGCAGTGCCCATATCGGTCCCCAGATACAAACATGTGGAGCAGAATGGTGAGAAGTTTGTG |
| hsa_circRNA_102032 | CTAGAGTTGGGAAGACATCACTGATTATGTCTCTGGTCAGTGAAGAATTTCCAGAAGAGGTTCCTCCCCGGGCAGAAGAAATCACCATTCCAGCTGATGTCACCCCAGAGAGAGTTCCAACACACATTGTAGATTACTCAGAAGCAGAACAGAGTGATGAACAACTTCATCAAGAAATATCTCAGGCTAATGTCATCTGTATAGTGTATGCCGTTAACAACAAGCATTCTATTGATAAGGTAACAAGTCGATGGATTCCTCTCATAAATGAAAGAACAGACAAAGACAGCAG |
