# Supplementary material for: A computational model of circRNA-associated diseases based on a graph neural network: prediction and case studies for follow-up experimental validation
Source: BMC Biol. 2024 Jan 29;22:24. doi: 10.1186/s12915-024-01826-z (PMC10823650; doi:10.1186/s12915-024-01826-z)
Supplement: Supplementary file 2 — Additional file 2: Table S2. The primer sequences of 10 circRNAs. [file 12915_2024_1826_MOESM2_ESM.docx]

Table S2. The primer sequences of 10 circRNAs

| No. | circRNA name | Reversed primer | Forward primer |
| --- | --- | --- | --- |
| 1 | hsa_circRNA_104135 | GTCACAGATGCGAGAACACAGG | AGCCTGAACACACTGGGAAATG |
| 2 | hsa_circRNA_102347 | CCTTCCTCTTGCTGTTCTGTATGAC | AATGGTAGAGGATGCGTGTATGTTG |
| 3 | hsa_circRNA_400031 | GCTCTTCCTTGCCTCTGTGC | TGCGTATTATGAGGTGCCAAGAC |
| 4 | hsa_circRNA_103096 | GCCTCCTTCCACTTGAACTGC | TTGGATGACACCGAAGTTAAGAAGG |
| 5 | hsa_circRNA_103809 | CTGAACTGCCTGTAACTCCTCTTC | TGACGCTCTGGCTGCTCTAC |
| 6 | hsa_circRNA_100571 | GACTGTATCACTACGCCACCATTC | TTAGCCACTGGTCCTGTCCTG |
| 7 | hsa_circ_0002577 | CTGTCCCTTTGCCTTCCCATTC | TGTTATACGCAGCAGATACTTACGG |
| 8 | hsa_circRNA_100338 | TCGTCACTGGGATCTAGGTTATCTG | ATGTGGAGCAGAATGGTGAGAAG |
| 9 | hsa_circRNA_102032 | GCTGGAATGGTGATTTCTTCTGC | ATGAAAGAACAGACAAAGACAGCAG |
| 10 | hsa_circ_0000520 | CAGACCTTCCCAAGGGACAT | CTAACAGGGCTCTCCCTGAG |
